# Supplementary material for: A social engineering model for poverty alleviation
Source: Nat Commun. 2020 Dec 11;11:6345. doi: 10.1038/s41467-020-20201-4 (PMC7732988; doi:10.1038/s41467-020-20201-4)
Supplement: Supplementary file 1 — Supplementary Information [file 41467_2020_20201_MOESM1_ESM.pdf]

A social Engineering Model for Poverty Alleviation
ONLINE SUPPLEMENTARY

Chattopadhyay, et al

In order to establish the veracity of the methodology used, we have repeated the analysis using econometric data for US basic (total food) food consumption data obtained from the World Bank website `http://data.worldbank.org/country/united-states`, spanning 8 years between 2007-2014. The resultant comparative histograms are all enclosed in the appended plot. In this supplementary section, all plots will be enumerated as *Supplementary Figures*, entitled *Figure S1, Figure S2, Figure S3 and Figure S4.*

This shows how the probability density function evolves with time (Figure S1). Unlike in Figure 1 (inset) of the main manuscript, we have deliberately refrained from data collapsing the Probability Density Functions (PDFs) all on the same plot. This is to emphasize how the income distribution evolves with time.

A quantitative estimation of this is enclosed in Figure S2 and Figure S3, in the form of the Pareto scaling gradient measure. The plots shown in both figures reconfirm the quintessential Pareto scaling that the large income sector of the income distribution probability density function shows and how they evolve with time. This representative plot should already serve as a resounding confirmation of the ML strategy used. We intend to redo the entire analysis using all 6 ML algorithms in a separate publication.

Figure S4 depicts the time dynamics of the distribution functions, represented as an evolution of the Pareto gradient from the relationship: PDF$(y) \sim y^{-\theta}$, where y represents the income statistics and θ the Pareto gradient.

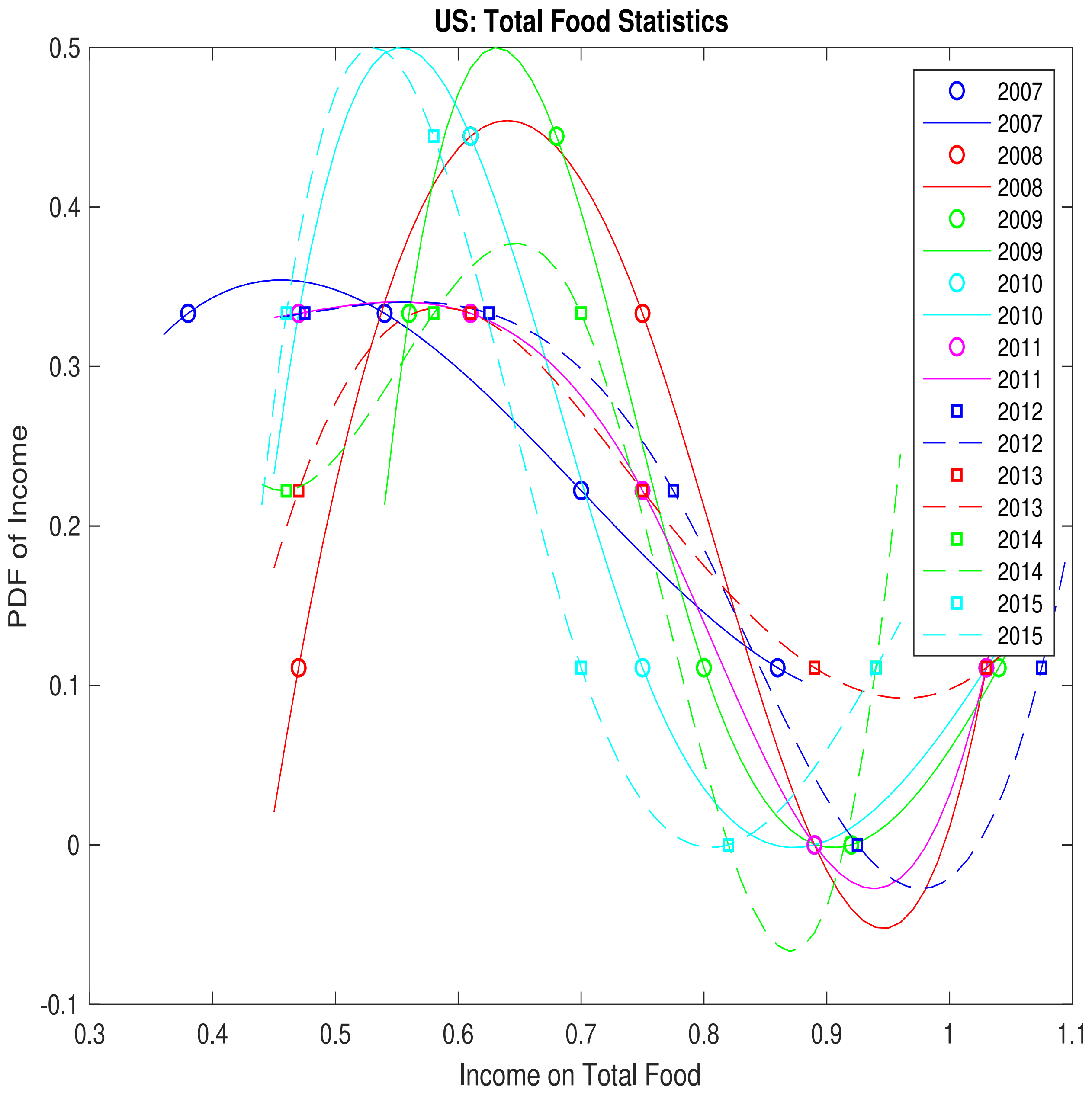

Figure S1: Time dynamics of histograms of the US total food consumption data

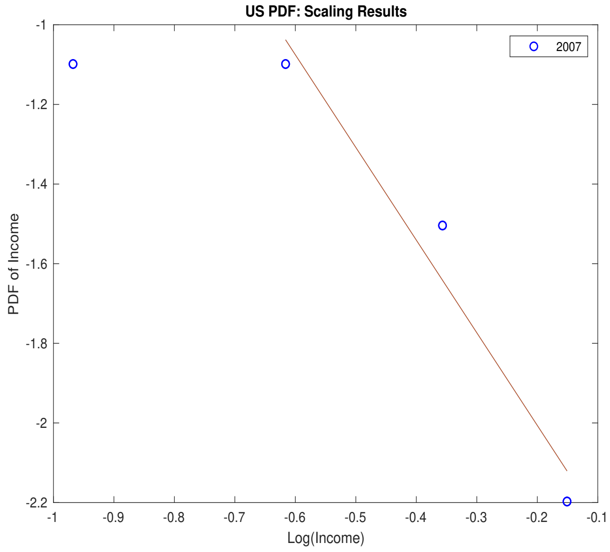

(a) 2007

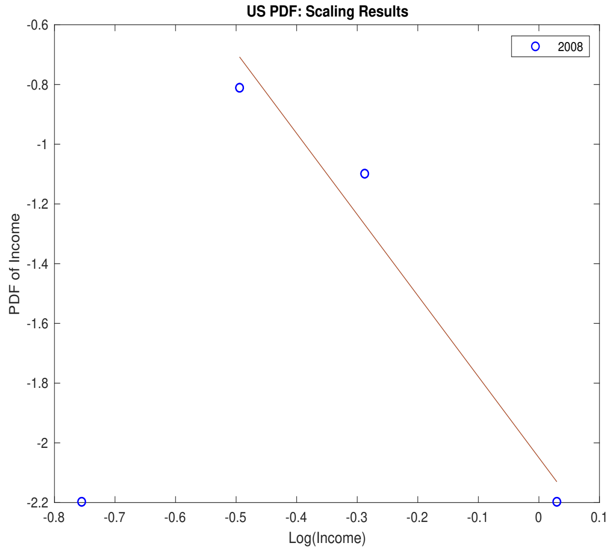

(b) 2008

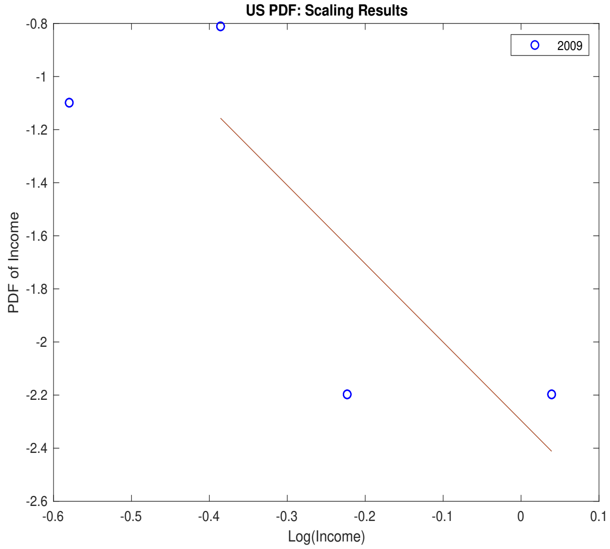

(c) 2009

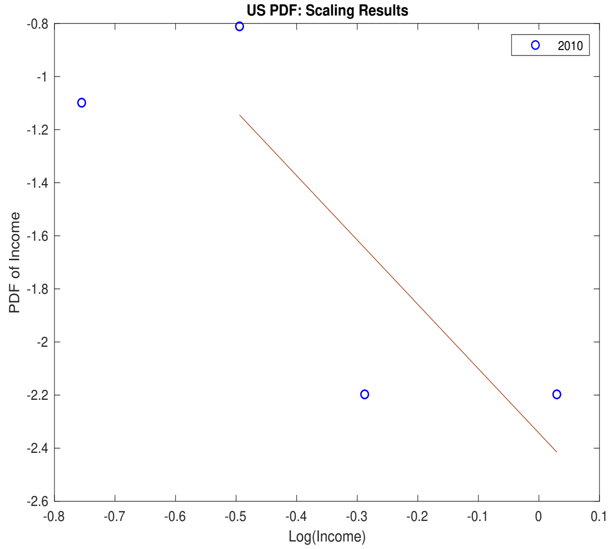

(d) 2010

Figure S2: Pareto scaling of PDF for large income in a log-log plot for years 2007, 2008, 2009, 2010

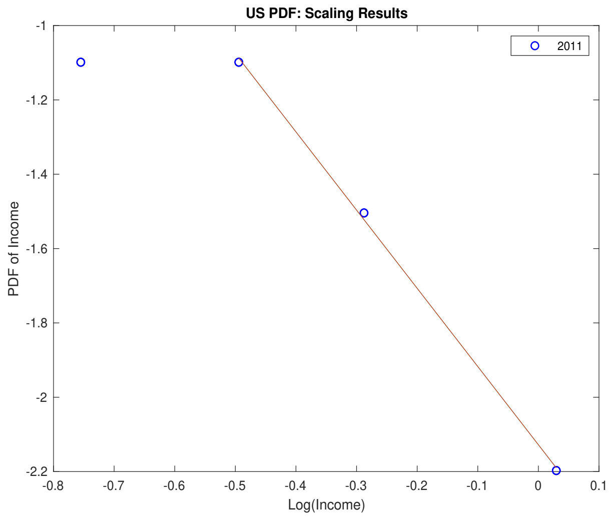

(a) 2011

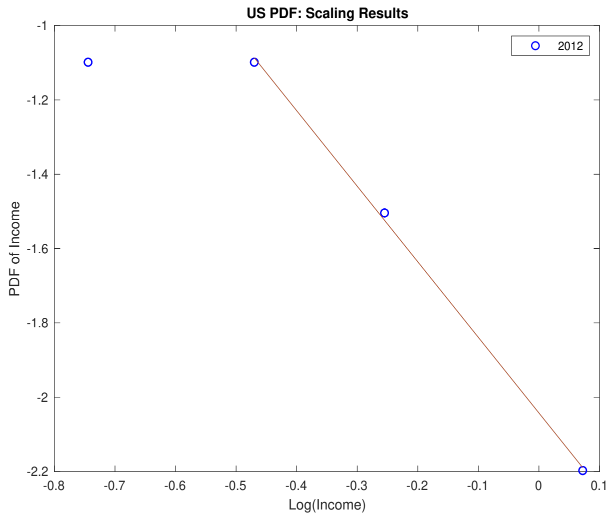

(b) 2012

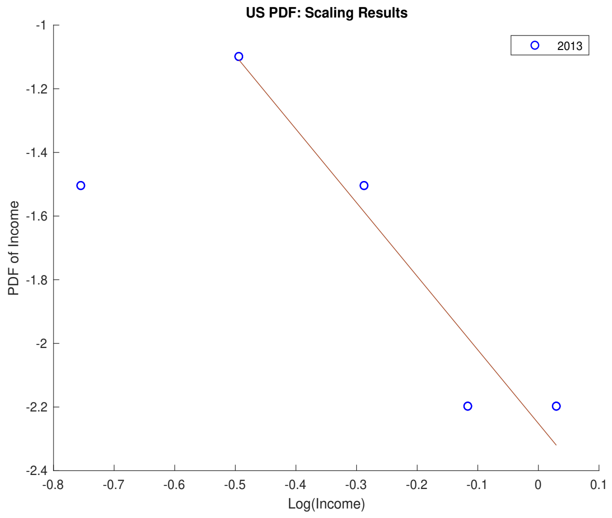

(c) 2013

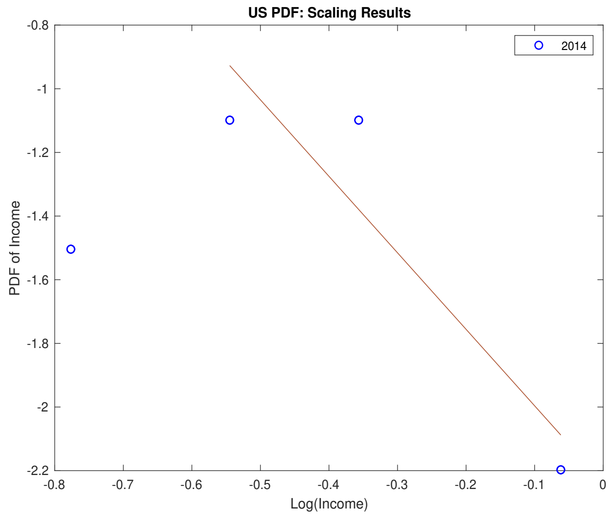

(d) 2014

Figure S3: Pareto scaling of PDF for large income in a log-log plot for years 2011, 2012, 2013, 2014

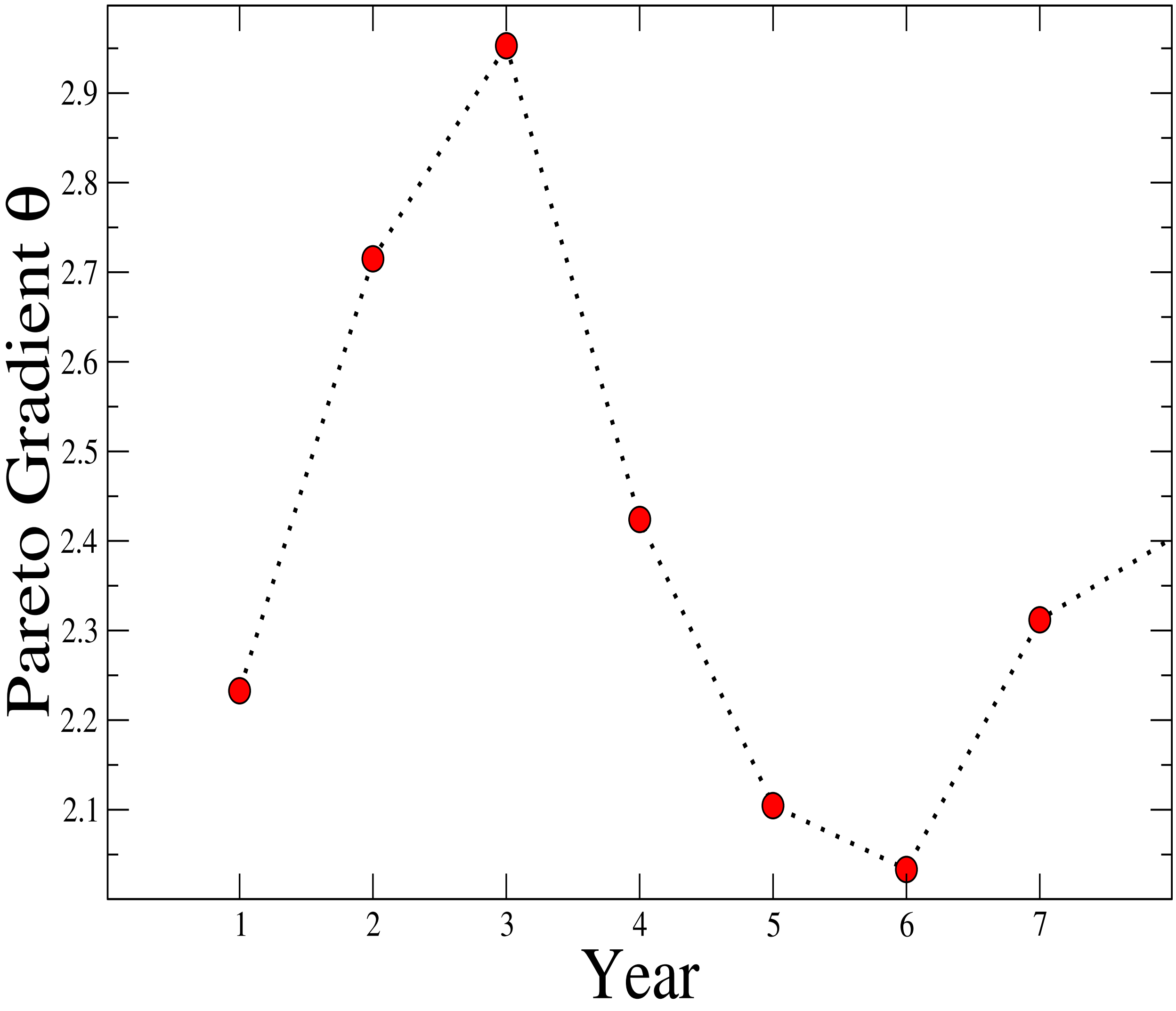

Figure S4: Time dynamics of the US total food (income)consumption statistics, quantified through the Pareto gradient (y-axis) plotted against the corresponding year number(x-axis), where “1” refers to 2007 and “8” refers to the year 2014.
